# Response to iron overload in cultured hepatocytes

**DOI:** 10.1038/s41598-020-78026-6

**Published:** 2020-12-03

**Authors:** Hsuan-Ju Chen, Makoto Sugiyama, Fumie Shimokawa, Masaru Murakami, Osamu Hashimoto, Tohru Matsui, Masayuki Funaba

**Affiliations:** 1 Division of Applied Biosciences, Graduate School of Agriculture, Kyoto University, Kitashirakawa Oiwakecho, Kyoto, 606-8502 Japan; 2 Laboratory of Veterinary Anatomy, Kitasato University School of Veterinary Medicine, Towada, 034-8628 Japan; 3 Laboratory of Molecular Biology, Azabu University School of Veterinary Medicine, Sagamihara, 252-5201 Japan; 4 Laboratory of Experimental Animal Science, Kitasato University School of Veterinary Medicine, Towada, 034-8628 Japan; 5 Present Address: Department of Animal Bioscience, Nagahama Institute of Bio-Science and Technology, Nagahama, 526-0829 Japan

**Keywords:** Cell growth, Mechanisms of disease

## Abstract

Iron is essential for a variety of physiological processes. Hepatic iron overload acts as a trigger for the progression of hepatic steatosis to nonalcoholic steatohepatitis and hepatocellular carcinoma. In the present study, we aimed to study the effects of iron overload on cellular responses in hepatocytes. Rat primary hepatocytes (RPH), mouse primary hepatocytes (MPH), HepG2 human hepatoma cells and Hepa1-6 mouse hepatoma cells were treated with FeCl_3_. Treatment with FeCl_3_ effectively increased iron accumulation in primary hepatocytes. Expression levels of molecules involved in cellular signaling such as AMPK pathway, TGF-β family pathway, and MAP kinase pathway were decreased by FeCl_3_ treatment in RPH. Cell viability in response to FeCl_3_ treatment was decreased in RPH but not in HepG2 and Hepa1-6 cells. Treatment with FeCl_3_ also decreased expression level of LC-3B, a marker of autophagy in RPH but not in liver-derived cell lines. Ultrastructural observations revealed that cell death resembling ferroptosis and necrosis was induced upon FeCl_3_ treatment in RPH. The expression level of genes involved in iron transport varied among different liver-derived cells- iron is thought to be efficiently incorporated as free Fe^2+^ in primary hepatocytes, whereas transferrin-iron is the main route for iron uptake in HepG2 cells. The present study reveals specific cellular responses in different liver-derived cells as a consequence of iron overload.

## Introduction

Iron is an essential element for all the living organisms, and is involved in a wide variety of physiological processes. However, excess iron has adverse effects on various organs through induction of oxidative stress^1–3^. Hepatic iron overload has been suggested to act as a trigger for the progression of hepatic steatosis to nonalcoholic steatohepatitis (NASH) and hepatocellular carcinoma^4,5^. Thus, the systemic iron levels should be strictly maintained. Since there is no particular regulatory route to excrete iron in mammals^6,7^, serum iron concentration should be maintained by the regulation of intestinal iron absorption and release via macrophages, the main storage system of body iron. The systemic iron status is controlled by hepcidin, which is a hormone produced by hepatocytes^7–9^. Increased synthesis and secretion of hepcidin in response to iron overload can stimulate internalization of ferroportin (Fpn), an iron exporter, leading to the inhibition of iron absorption by the intestines and further, the release via the macrophages^7–9^. Since Fpn is also expressed in hepatocytes, the hepatic Fpn can also be a target of hepcidin^10,11^.

Previous studies have shown that the treatment with non-transferrin bound iron (NTBI) resulted in alterations in cellular soundness in cultured liver-derived cells, including induction of cell death^12–16^. The NTBI-induced cell toxicity is linked to oxidative stress^16^. The treatment with ferric chloride (FeCl_3_) or iron citrate stimulated lipid peroxidation in rat or human primary hepatocytes, respectively, indicating onset of oxidative stress^12,13^. Treatment with hydrogen peroxide, an inducer of oxidative stress, led to necrosis of hepatocytes^17^. Further, FeCl_3_ enhanced hydrogen peroxide-induced cell death in rat primary hepatocytes (RPH)^18^. NTBI also increased cytosolic concentration of Ca^2+^ ^12^ and leakage of aspartate aminotransferase and alanine transaminase^13^ in primary hepatocytes. Cell viability decreased upon treatment with 100 μM FeCl_3_ in HepG2 human hepatoma cells, but no DNA damage was detected in cells treated with up to 800 μM of FeCl_3_^15^.

The objective of this study is re-characterization of cell responses to FeCl_3_ treatment in cultured liver-derived cells. We evaluated cell viability/death, morphological features, expression of molecules related to cell signaling, modulation of gene expression in an early phase of FeCl_3_ treatment, and mRNA expression levels of molecules related to iron transport using primary hepatocytes as well as liver-derived cell lines.

## Results

### Excess uptake of iron induces cell death in primary hepatocytes

We first analyzed the effects of iron treatment in RPH (Supplementary Fig. 1). Upon treatment the RPH with FeCl_3_ at a concentration of 100 μM for 16 h, we observed a clear Berlin blue staining, a staining method used for detecting Fe^3+^ (Supplementary Fig. 1A). Similarly, MitoFerroGreen staining revealed that the treatment with FeCl_3_ led to an increase in the fluorescence intensity of the RPH (Supplementary Fig. 1B), indicating an increase in the level of mitochondrial Fe^2+^. These results suggest that FeCl_3_ is efficiently incorporated into RPH, leading to iron accumulation. Iron overload decreases the stability of Tfrc (transferrin type I receptor) mRNA through an iron-response element, which resides at the 3′-UTR of this gene^19^. The expression level of *Tfrc* was significantly decreased in the RPH upon treatment with FeCl_3_ (Supplementary Fig. 1C). As described above, iron overload is shown to induce oxidative stress^3^. The expression level of *Hmox1*, a gene known to increase in response to induction of oxidative stress^20^, was expectedly increased in RPH that were treated with FeCl_3_ (Supplementary Fig. 1D). Iron accumulation in response to FeCl_3_ treatment was also verified by ultrastructural analysis. The observation made by using transmission electron microscope (TEM) showed granules with high density of electron concentration in FeCl_3_-treated cells but not in control cells, suggesting the presence of iron granules (Supplementary Fig. 2). We also analyzed the mRNA level of *Tfrc* in mouse primary hepatocytes (MPH, Supplementary Fig. 3). Consistent with the results in RPH, *Tfrc* mRNA level was also significantly decreased upon FeCl_3_ treatment in MPH. This decrease in *Tfrc* mRNA level was detected upon FeCl_3_ treatment at a minimal concentration of 25 μM.

**Figure 1:**
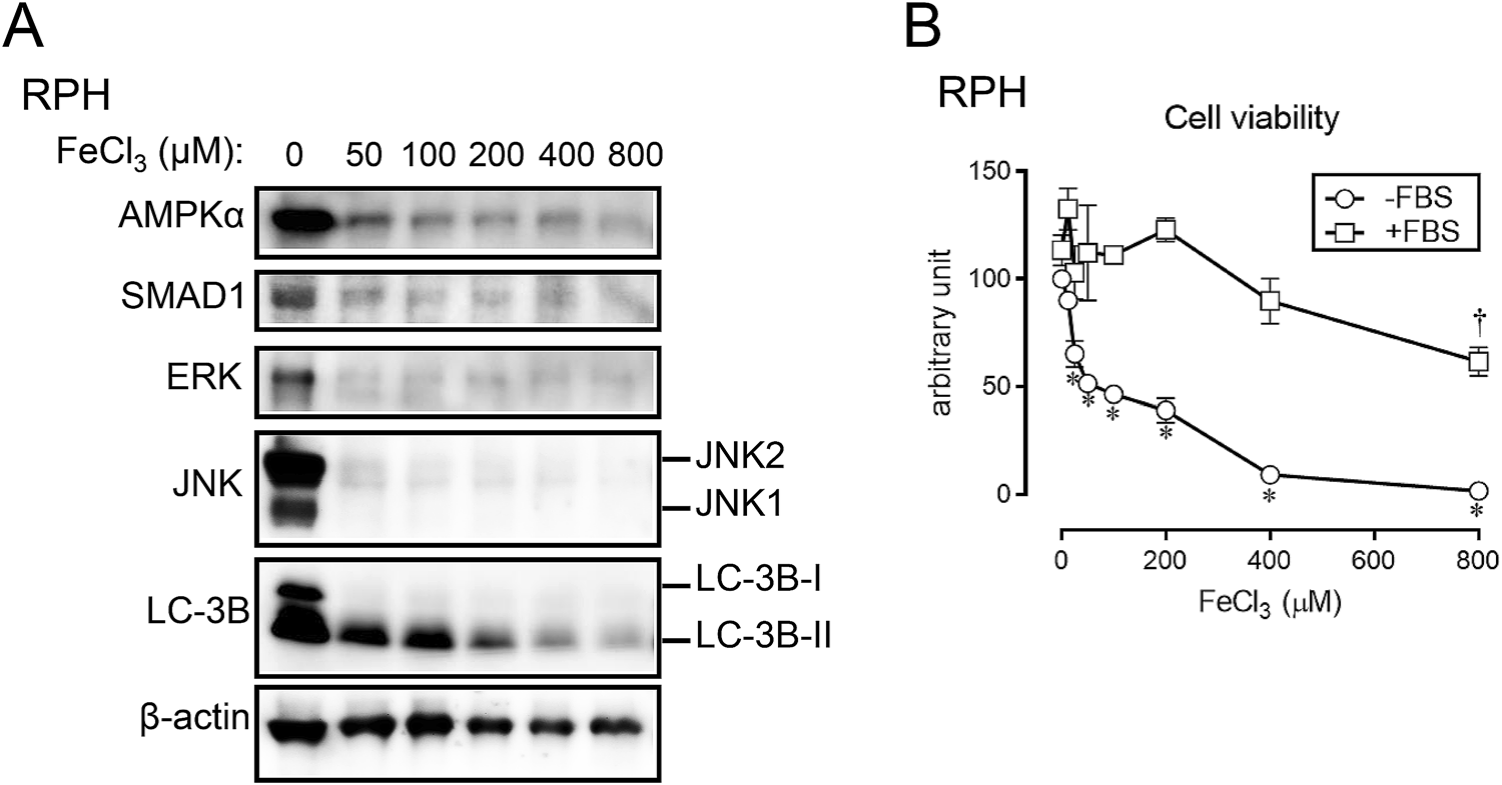
Iron overload decreases the protein expression level of several signaling molecules and the cell viability in RPH. (**A**) RPH were treated with the indicated concentration of FeCl_3_ for 24 h. Expression level of AMPKα, SMAD1, ERK, JNK, LC-3B, and β-actin was evaluated by Western blot analysis. (**B**) RPH were treated with the indicated concentration of FeCl_3_ in the presence or absence of FBS for 24 h, and thereafter the cell viability was measured. The cell viability of untreated control cells was set at 100. * and †: significantly decrease (*P* < 0.05), as compared with cells treated without FeCl_3_ in the absence of FBS and presence of FBS, respectively.

To explore how primary hepatocytes respond to iron overload, we examined the protein expression of molecules related to cell signaling: components involved in AMP kinase pathway, TGF-β family pathway, and MAP kinase pathway (Fig. 1A, Supplementary Fig. 4). In general, the expression level of these proteins tended to be decreased upon FeCl_3_ treatment (Supplementary Fig. 5). We also evaluated the expression level of LC-3B, a maker of autophagy. It has been previously shown that upon the onset of autophagy, LC-3-I is converted to LC-3-II, and LC-3-II disappears with the progression of autophagy^21^. The expression level of LC-3B-I as well as LC-3B-II decreased in primary hepatocytes upon treatment with increasing concentration of FeCl_3_.

Previous studies have shown that treatment with excess NTBI induced cell death in cultured hepatocytes^16,18^. We next evaluated cell viability in RPH (Fig. 1B). The cells were treated with different concentration of FeCl_3_ for 24 h, and to assess the cell viability we evaluated the cellular ATP content. When cells were treated with FeCl_3_ in the absence of FBS, the cell viability was found to decrease in a dose-dependent manner; the IC_50_ was estimated to be 63.8 μM. Contrastingly, the reduction in cell viability was not detected, when the cells were treated with FeCl_3_ in the presence of FBS. It has been shown that Fe^3+^ is adsorbed by molecules (presumably albumin) in FBS, and therefore it is possible that the FeCl_3_ treatment in this case did not induce cell death.

**Figure 2:**
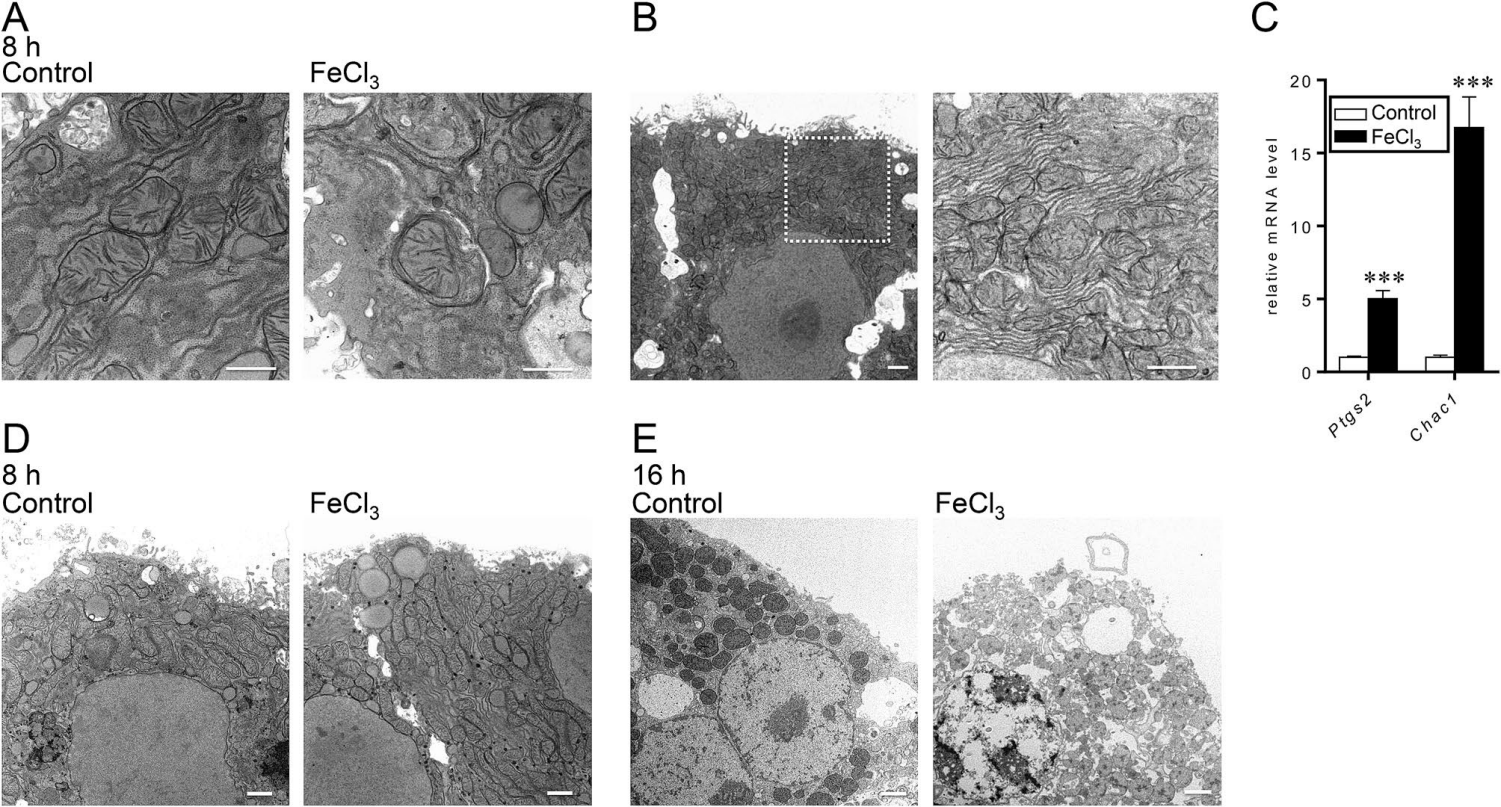
RPH exhibit morphological features such as mitophagy, ferroptosis, and necrosis. RPH were treated with FeCl_3_ (0 or 100 μM) for 8 h or 16 h. Ultrastructure of these hepatocytes was analyzed by transmission electron microscopy. (**A**) Representative images depicting the autophagy/mitophagy phenotype in RPH treated with FeCl_3_ (*right panel*) for 8 h but not control RPH (*left panel*). The bar in the image indicates 500 nm. (**B**) Representative image of FeCl_3_-treated RPH depicting the increase in electric density of cytoplasm and mitochondria (*left panel*), and higher magnification of the dotted line square (*right panel*). The bar in the image indicates 1 μm. (**C**) Expression level of *Ptgs2* and *Chac1* genes was examined by RT-qPCR analysis. The expression level of these genes in control RPH was set at 1. ***P < 0.001. (**D**,**E**) Representative images of the necrotic features of RPH treated with FeCl_3_ (*right panel*) for 8 h (**D**) or 16 h (**E**) but not control RPH (*left panel*) are presented. The bar in the image is indicates 1 μm.

Observation of the cell ultrastructure revealed that the RPH treated with FeCl_3_ for 8 h but not the control RPH exhibited phagophore surrounding the mitochondrion, suggesting the onset of mitophagy, which is reported as a kind of autophagy^22^ (Fig. 2A). Similar morphological features were detected in the primary hepatocytes treated with FeCl_3_ for 16 h (data not shown).

Since the cell viability assay indicated the induction of cell death in FeCl_3_-treated primary hepatocytes, we speculated that the cell death is related to ferroptosis, cell death induced by iron-dependent peroxidized lipids^23^. Ultrastructural analyses of cell morphology indicated that apoptotic cells with condensed chromatin were not observed upon FeCl_3_ treatment (data not shown). In some hepatocytes, the nuclear size was unchanged and the integrity of cytoplasmic membrane was well maintained (Fig. 2B, *left*), but the electric density of cytoplasm and mitochondria was increased (Fig. 2B, *left*). Furthermore, the outer membrane of some mitochondrion disappeared and the mitochondria christae were contracted (Fig. 2B, *right*). These features clearly resemble to the features of ferroptotic cells^24,25^. Treatment with FeCl_3_ up-regulated the expression level of *Ptgs2* and *Chac1* genes (Fig. 2C); the expression levels of these genes are known to increase in ferroptotic cells^26^. These results suggest that RPH undergo ferroptosis upon treatment with FeCl_3_.

We also observed FeCl_3_-treated cells with disappeared cytoplasmic membrane and enlarged mitochondria, whereas these features were not detected in control cells (Fig. 2D). Further, some cells exhibited loss of cytoplasm, a feature that resembles necrosis/necroptosis of FeCl3-treated hepatocytes (Fig. 2E). All these results suggest that the primary hepatocytes treated with FeCl_3_ undergo cell death through ferroptosis and necrosis/necroptosis.

### Excess FeCl_3_ rapidly modulates expression level of genes in RPH

**Figure 3:**
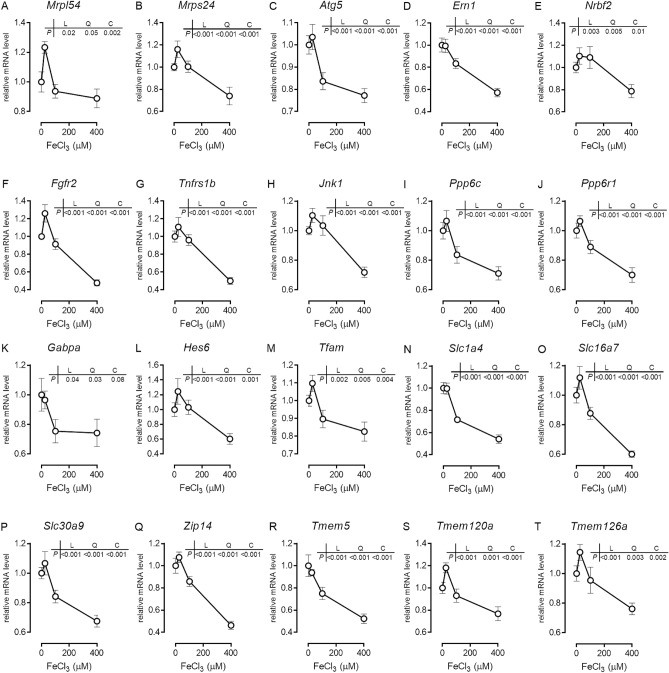
Expression level of genes in several categories rapidly change in response to FeCl_3_ treatment in RPH. RPH were treated with the indicated concentration of FeCl_3_ for 2 h. The expression level of genes related to (**A**,**B**) ribosomal proteins, (**C**–**E**) autophagy/stress-related, (**F**–**H**) cell signaling, (**I**,**J**) protein phosphatase, (**K**-**M**) transcription factors, (**N**-**Q**) transporters, and (**R**-**T**) transmembrane proteins were examined by RT-qPCR analysis. The expression level of all these genes in the control untreated cells was set at 1.

We next investigated the early events of FeCl_3_-induced cell response. We performed a comprehensive RNA-seq analyses using RPH treated with FeCl_3_ for 2 h. We then performed the Gene Ontology (GO) analyses for the genes, which exhibited alterations in the expression level upon treatment with FeCl_3_ in a dose-dependent manner. Ribosome- and nucleosome-related genes showed significant alterations in their expression level (Table 1). We also quantified the expression level of several genes out of the 174 FeCl_3_-responsive genes that were found by RNA-Seq analyses. The list included the genes related to ribosomal proteins, autophagy/stress, cell signaling, protein phosphatases, transcription factors, transporters, and transmembrane proteins (Fig. 3). Expression level of the tested genes exhibited quadratic or cubic changes against the concentration of FeCl_3_; expression levels of multiple molecules tended to be higher in cells treated with 25 μM FeCl_3_ than in control cells. This may reflect resistance to iron overload. In contrast, the expression level of these genes in cells treated with 800 μM FeCl_3_ was significantly lower than those in control cells, which is likely to relate to response toward cell death. The expression level of these genes was basically interrelated (Table 2). These results suggest that FeCl_3_ treatment equally modulates the expression of genes in several categories, which might also be responsible for FeCl_3_-induced cell death in RPH.

**Table 1:** Summary of the descriptive GO term names of the functional clusters affected by FeCl_3_.

Category	Enrichment score
Ribosome	7.74
Nucleosome	2.80
Isopeptide bond/methylation	1.85
NAFLD and other diseases	1.35

Using the DAVID functional annotation clustering analysis, genes modulated by FeCl_3_ in a dose-dependent manner were clustered according to GO terms. A summarized description of the significant clusters and enrichment score is shown.

**Table 2:** Relationship between expression levels of genes affected by FeCl3 in rat primary hepatocytes.

		Ribosomal protein		Autophagy/stress-related			Cell signaling			Protein phosphatase		Transcription factor			Transporter				Transmembrane protein		
		Mrpl54	Mrps24	Atg5	Ern1	Nrbf2	Fgfr2	Tnfrs1b	Jnk1	Ppp6c	Ppp6r1	Gabpa	Hes6	Tfam	Slc1a4	Slc16a7	Slc30a9	Zip14	Tmem5	Tmem120a	Tmem126a
Ribosomal protein	Mrpl54	–	0.774	0.735	0.703	0.669	0.678	0.649	0.530	0.782	0.636	0.561	0.671	0.764	0.721	0.726	0.691	0.676	0.707	0.855	0.827
			< 0.001	< 0.001	< 0.001	< 0.001	< 0.001	< 0.001	0.008	< 0.001	< 0.001	0.004	< 0.001	< 0.001	< 0.001	< 0.001	< 0.001	< 0.001	< 0.001	< 0.001	< 0.001
	Mrps24		–	0.725	0.839	0.795	0.826	0.830	0.725	0.802	0.805	0.579	0.888	0.782	0.838	0.839	0.855	0.869	0.722	0.899	0.791
				< 0.001	< 0.001	< 0.001	< 0.001	< 0.001	< 0.001	< 0.001	< 0.001	0.003	< 0.001	< 0.001	< 0.001	< 0.001	< 0.001	< 0.001	< 0.001	< 0.001	< 0.001
Autophagy/stress-related	Atg5			–	0.841	0.591	0.750	0.680	0.607	0.783	0.793	0.574	0.631	0.708	0.783	0.806	0.783	0.723	0.726	0.728	0.739
					< 0.001	0.003	< 0.001	< 0.001	0.002	< 0.001	< 0.001	0.004	0.001	< 0.001	< 0.001	< 0.001	< 0.001	< 0.001	< 0.001	< 0.001	< 0.001
	Ern1				–	0.769	0.912	0.900	0.761	0.858	0.853	0.643	0.831	0.699	0.897	0.936	0.817	0.933	0.824	0.814	0.820
						< 0.001	< 0.001	< 0.001	< 0.001	< 0.001	< 0.001	< 0.001	< 0.001	< 0.001	< 0.001	< 0.001	< 0.001	< 0.001	< 0.001	< 0.001	< 0.001
	Nrbf2					–	0.735	0.801	0.530	0.653	0.607	0.383	0.716	0.441	0.624	0.736	0.624	0.723	0.526	0.752	0.773
							< 0.001	< 0.001	0.008	< 0.001	0.002	0.07	< 0.001	0.03	0.001	< 0.001	0.001	< 0.001	0.008	< 0.001	< 0.001
Cell signaling	Fgfr2						–	0.929	0.845	0.839	0.802	0.451	0.812	0.720	0.885	0.936	0.831	0.935	0.808	0.842	0.819
								< 0.001	< 0.001	< 0.001	< 0.001	0.03	< 0.001	< 0.001	< 0.001	< 0.001	< 0.001	< 0.001	< 0.001	< 0.001	< 0.001
	Tnfrs1b							–	0.780	0.809	0.740	0.373	0.784	0.662	0.855	0.887	0.809	0.918	0.776	0.815	0.812
									< 0.001	< 0.001	< 0.001	0.07	< 0.001	< 0.001	< 0.001	< 0.001	< 0.001	< 0.001	< 0.001	< 0.001	< 0.001
	Jnk1								–	0.726	0.830	0.510	0.714	0.761	0.721	0.816	0.803	0.835	0.806	0.656	0.637
										< 0.001	< 0.001	0.01	< 0.001	< 0.001	< 0.001	< 0.001	< 0.001	< 0.001	< 0.001	< 0.001	< 0.001
Protein phosphatase	Ppp6c									–	0.739	0.664	0.732	0.870	0.867	0.876	0.880	0.850	0.809	0.831	0.853
											< 0.001	< 0.001	< 0.001	< 0.001	< 0.001	< 0.001	< 0.001	< 0.001	< 0.001	< 0.001	< 0.001
	Ppp6r1										–	0.659	0.770	0.786	0.837	0.839	0.872	0.862	0.817	0.747	0.668
												< 0.001	< 0.001	< 0.001	< 0.001	< 0.001	< 0.001	< 0.001	< 0.001	< 0.001	< 0.001
Transcription factor	Gabpa											–	0.570	0.646	0.591	0.580	0.594	0.568	0.539	0.518	0.533
													0.004	< 0.001	0.002	0.003	0.002	0.004	0.007	0.01	0.007
	Hes6												–	0.694	0.779	0.796	0.747	0.806	0.680	0.762	0.713
														< 0.001	< 0.001	< 0.001	< 0.001	< 0.001	< 0.001	< 0.001	< 0.001
	Tfam													–	0.798	0.727	0.888	0.749	0.814	0.790	0.771
															< 0.001	< 0.001	< 0.001	< 0.001	< 0.001	< 0.001	< 0.001
Transporter	Slc1a4														–	0.897	0.911	0.926	0.865	0.864	0.788
																< 0.001	< 0.001	< 0.001	< 0.001	< 0.001	< 0.001
	Slc16a7															–	0.857	0.939	0.828	0.821	0.784
																	< 0.001	< 0.001	< 0.001	< 0.001	< 0.001
	Slc30a9																–	0.866	0.814	0.808	0.798
																		< 0.001	< 0.001	< 0.001	< 0.001
	Slc39a14																	–	0.865	0.843	0.789
																			< 0.001	< 0.001	< 0.001
Transmembrane protein	Tmem5																		–	0.758	0.726
																				< 0.001	< 0.001
	Tmem120a																			–	0.856
																					< 0.001
	Tmem126a																				–

Upper: correlation coefficient. Lower: *P* value.

### Liver-derived cell lines are resistant to the effects of excess FeCl_3_

**Figure 4:**
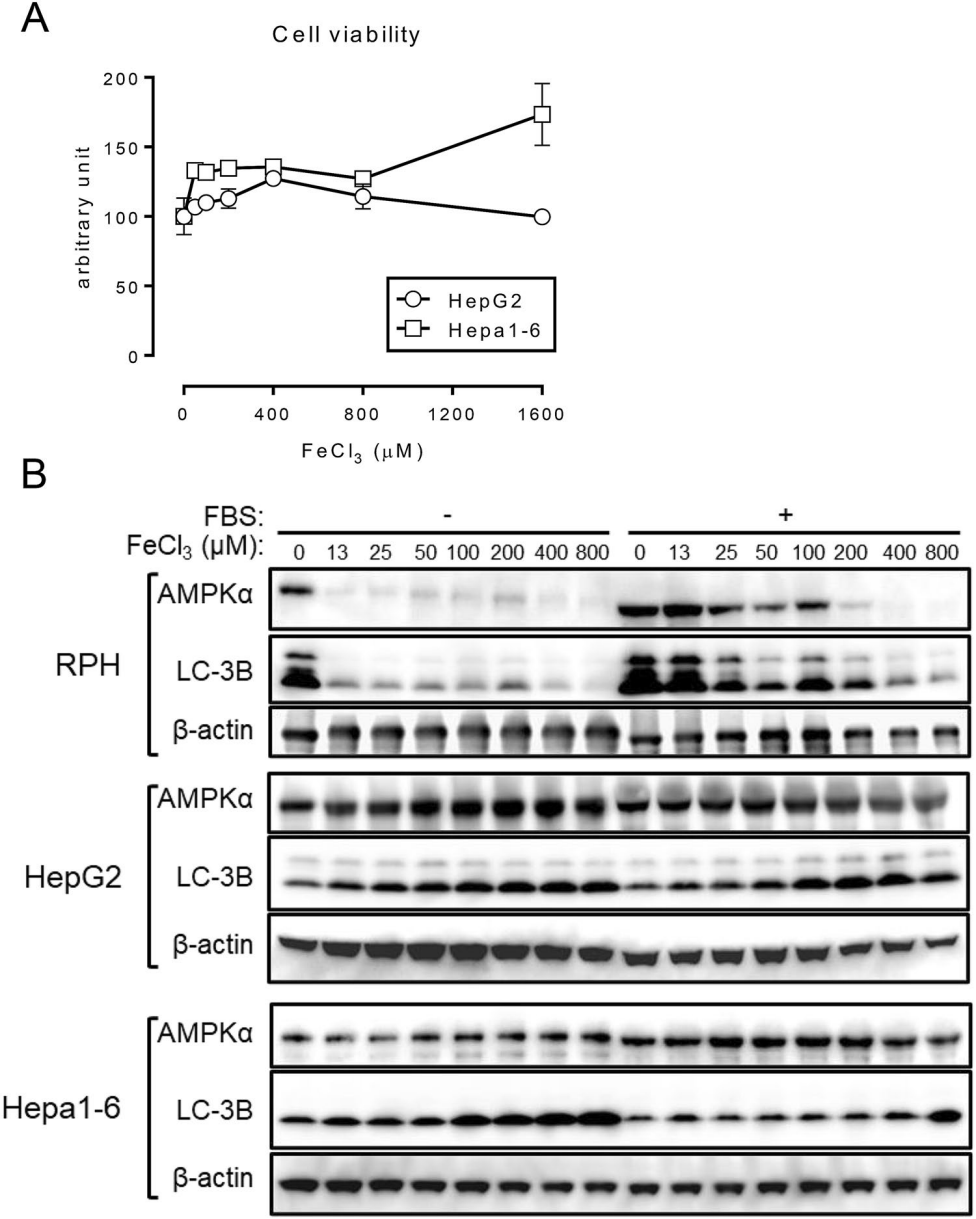
Hepatic cell lines do not undergo cell death and also do not show a decrease in AMPK and LC-3B protein levels in response to FeCl_3_ treatment. (**A**) HepG2 cells and Hepa1-6 cells were treated with the indicated concentration of FeCl_3_ in the absence of FBS for 24 h, and thereafter the cell viability was measured. Cell viability of untreated cells was set at 100. (**B**) RPH, HepG2 cells, and Hepa1-6 cells were treated with different concentration of FeCl_3_ (0, 12.5, 25, 50, 100, 200, 400, or 800 μM) for 24 h. Expression of AMPKα, LC-3B, and β-actin was evaluated using Western blot analysis.

We examined whether FeCl_3_-induced cell death also occurs in other liver-derived cell lines, such as HepG2 and Hepa1-6 in the absence of FBS (Fig. 4A). The decrease in cell viability in response to FeCl_3_ treatment was not evident in these liver-derived cell lines. In addition, the decreased expression of AMPKα in response to FeCl_3_ treatment in RPH (Fig. 1A) was not detected in HepG2 and Hepa1-6 cells (Fig. 4B, Supplementary Figs. 6 and 7); FeCl_3_ treatment reproducibly decreased AMPKα expression, irrespective of the presence of FBS. Further, treatment with FeCl_3_ did not affect the expression level of LC-3B in the liver-derived cell lines, which was detected even in cells cultured in the absence of FBS (Fig. 4B, Supplementary Figs. 6 and 7).

In order to understand the reason of this cell-dependent resistance to FeCl_3_, we analyzed the expression level of molecules related to iron uptake and export. Iron is incorporated into the cells after reduction or upon binding to transferrin as Fe^3+^. In hepatocytes, Dctyb and Steap4 are well-known reductases of Fe^3+^ that are present on the cell membrane, and Zip14 as a transporter of Fe^2+^ ^27–30^. Transferrin-bound iron uptake is also reported to be mediated through Tfrc. The transferrin-bound iron is first internalized, and then reduced by Steap3 in the endosome, and at last transferred to the cytosol via Dmt1^31,32^. Inorganic Fe^2+^ in cytoplasm is exported from cytoplasm via Fpn. Alternatively, excess iron is stored as Fe^3+^-ferritin complex^31,32^. Iron in hepatocytes also exists in the form of a complex with heme^31,32^. Heme iron is released through breakdown of heme by Hmox1. Thus, we compared the expression level of *Dcytb*, *Steap4*, *Zip14*, *Tfrc*, *Steap3*, *Dmt1*, *Fpn*, ferritin heavy chain (*Fth1*), and *Hmox1* among RPH, MPH, HepG2, and Hepa1-6 cells. Since the origin of liver-derived cells was different (HepG2 and Hapa1-6 cells were derived from the liver of human and mouse, respectively), common primers to detect the corresponding genes from human, mouse and rats were prepared (Supplementary Table 1). The amplification efficiency was comparable among genes of different species; a part of results is shown in Supplementary Fig. 8.

**Figure 5:**
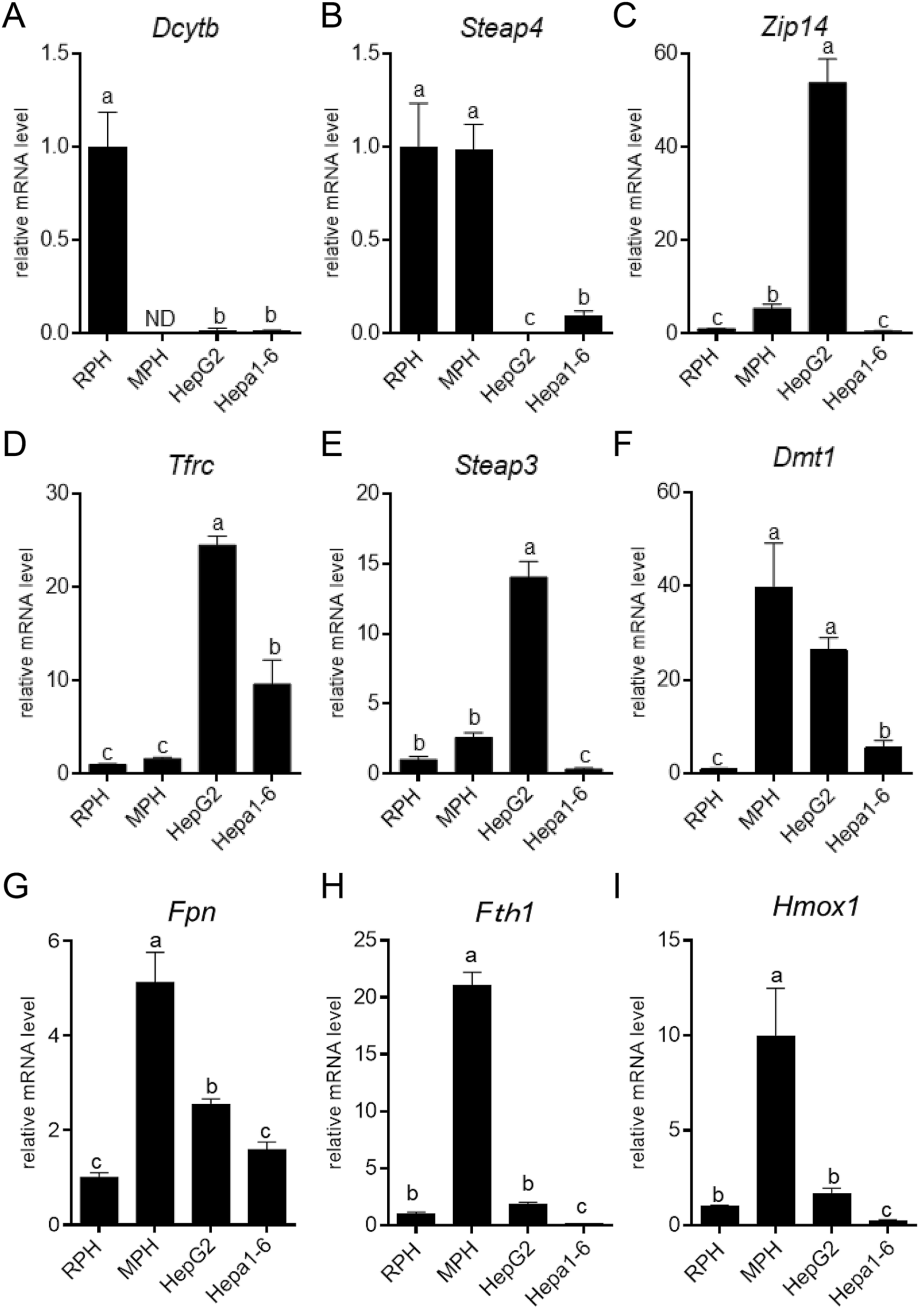
Expression level of genes related to iron metabolism is different among liver-derived cell types. Expression level of genes related to iron transport was quantified in RPH, MPH, HepG2 cells, and Hepa1-6 cells. Expression level of (**A**) *Dcytb*, (**B**) *Steap4*, (**C**) *Zip14*, (**D**) *Tfrc*, (**E**) *Steap3*, (**F**) *Dmt1*, (**G**) *Fpn*, (**H**) *Fth1*, and (**I**) *Hmox1* was examined in RPH, MPH, HepG2 cells and Hepa1-6 cells by RT-qPCR using the common primers for respective genes from human, mouse, and rat. Expression level in RPH was set at 1. The letters a, b, and c indicate that the mean is significantly different (*P* < 0.05). ND indicates that the transcript was not detected.

The expression level of *Dcytb* was higher in RPH than in other cells (Fig. 5A). The expression level of *Steap4* was also higher in RPH than in HepG2 or Hepa1-6 cells (Fig. 5B). *Steap4* expression level was also higher in MPH. The expression level of *Zip14*, *Tfrc*, and *Steap3* was higher in HepG2 cells than in Hepa1-6 cells and primary hepatocytes (Fig. 5C–E). *Dmt1* expression level was higher in MPH and HepG2 cells (Fig. 5F). The expression level of *Fpn*, *Fth1*, and *Hmox1* was higher in MPH than in other cells (Fig. 5G–I).

## Discussion

Here, we re-characterized the response to iron overload in cultured liver-derived cells. Previous studies have shown that NTBI induced alterations in cellular soundness in cultured liver-derived cells, leads to a decrease in cell viability^12–16^. In the present study, we revealed that the RPH morphologically undergoes ferroptosis and necrosis/necroptosis in response to FeCl_3_ overload, whereas liver-derived cell lines exhibit resistance to FeCl_3_-induced cell death. The differential expression level of molecules related to iron transport might be involved in this cell type-dependent regulation. Our results suggest the use of suitable liver-derived cells depending on the objective of the study.

Considering that expression levels of reductase on the cell membrane were relatively higher in primary hepatocytes (Fig. 5A,B), Fe^3+^ is likely to be efficiently reduced on the cell membrane in these hepatocytes. In contrast, HepG2 and Hepa1-6 cells are hardly able to convert Fe^3+^ to Fe^2+^. Therefore, iron may not be able to incorporate into the cell via Zip14 in liver-derived cell lines, even if expression level of *Zip14* was relatively higher in HepG2 cells (Fig. 5C). In HepG2 cells, uptake as transferrin-iron may be the main route, because mRNA levels of *Tfrc*, *Steap3* and *Dmt1* were relatively higher (Fig. 5D,E). The mRNA level of iron uptake and reduction enzyme was generally lower in Hepa1-6 cells, which may lead to a lower uptake of iron, irrespective of higher concentration of Fe^3+^ in the medium.

Extent of differentiation as the hepatocytes may be also involved in differential response to FeCl_3_ treatment between liver-derived cell lines and primary hepatocytes. Hep3B, more differentiated liver-derived cells as compared with HepG2, accumulated more iron in response to NTBI treatment^33^. It is possible that expression levels of molecules related to iron transport in Hep3B cells exhibit similar pattern to those in primary hepatocytes. The present study evaluated the expression at the mRNA level of molecules related to iron transport in cultured cells. Future studies are needed to evaluate the expression level at the protein level in various liver-derived cells. Further, dynamics of iron as well as cell death should be clarified in future.

Our results demonstrate that HepG2 cells are resistant to FeCl_3_ for cell viability, which is contrasting to the results of the study by Terpilowska et al*.*^15^, who showed a decrease in the cell viability in response to FeCl_3_. We suspect that the variable culture conditions might be responsible for these inconsistent results. In the present study, we cultured the HepG2 cells in the absence of FBS to evaluate cell viability, because the effect of FeCl_3_ treatment was masked in the presence of FBS in RPH. In contrast, in the study of Terpilowska et al.^15^, they assessed the effect of FeCl_3_ on cell viability in the presence of FBS. HepG2 cells actively proliferate in the presence of FBS. It is possible that the effects of FeCl_3_ just reflect the inhibition of cell growth rather than a decrease in cell viability in the presence of FBS. Alternatively, we evaluated the cell viability by measuring cellular ATP content, whereas Terpilowska et al.^15^ assessed the reduction of MTT (3-(4,5-dimethylthiazol-2-yl)-2,5-diphenyltetrazolium bromide), neutral red uptake, and release of lactate dehydrogenase. Estimates are likely to lead to differential suggestions.

RNA-seq analyses was performed to explore the early events in response to iron overload, which indicated that the expression level of many genes that are related to ribosomal proteins, autophagy/stress, mitochondrial respiratory chain, cell signaling, protein phosphatases, transcription factors, transporters, and transmembrane proteins was significantly altered. Changes in expression level of *Atg5*, *Jnk1*, and *Tfam* upon iron overload might be related to the onset of ferroptosis, because these gene products are involved in induction of ferroptosis^34–36^. However, the pattern of changes in the gene expression levels in response to iron overload was different in the present study; the expression levels decreased upon treatment with increasing concentration of FeCl_3_. The expression level of these genes might also change with time upon FeCl_3_ treatment, which was not assessed in this study.

Iron overload likely affects the metabolism of other minerals. The metabolism of other secondary minerals is known to be regulated through agonistic or antagonistic interactions between the minerals^37,38^. For instance, considering the expression level of *Zip14* (an importer of zinc as well as iron) and *Slc30a9* (an exporter for zinc), which decreased upon treatment with an increasing concentration of FeCl_3_ (Fig. 3P,Q), it is possible that iron overload will likely modulate zinc metabolism in hepatocytes. In fact, it has previously been shown that high iron intake leads to an increase in zinc accumulation in rat liver^39^; however, hepatic expression of *Zip14* was not affected by the high iron intake^39^. Also, it has been shown that Ga(NO_3_)_3_ and Al_2_(SO_4_)_3_ enhances NTBI uptake^40^. Cerulloplasmin, a major copper-carrying protein in the blood, is reported to stimulate NTBI uptake in liver cells^41,42^. The status of minerals other than iron might be different among liver-derived cells, which might lead to distinct cell responses to FeCl_3_ treatment.

In the present study, we tried to evaluate the direct effects of iron in hepatocytes. However, we also need to consider that the response to iron overload in hepatocytes might be modulated by liver resident cells, such as, Kupffer cells, endothelial cells and stellate cells. For example, it has previously been shown that sinusoidal cells can sense excess iron and produce BMP6, which further leads to an increased production of hepcidin in hepatocytes^43^. In addition, secretion of interleukin-1β, interleukin-6 as well as activin B in response to inflammation in Kupffer cells and sinusoidal cells have also been shown to induce hepcidin in hepatocytes^44,45^. Furthermore, we have previously shown that metabolic modulations in the liver in response to magnesium deficiency are triggered by the changes in endothelial cells^46^. Thus, these interactive effects among liver-resident cells in response to iron overload should be addressed in the future studies.

## Materials and methods

### Cell culture conditions

All procedures for the use of animal material were approved by the Kyoto University Animal Experiment Committee (29–20 and 30–20), and all animal experiments were conducted in accordance with the approved guidelines. Primary hepatocytes from the livers of 4-week-old male Sprague–Dawley rats or 5–8 weeks old male ICR mice were isolated as described previously^44,47^. RPH, MPH, HepG2 human hepatoma cells, and Hepa1-6 mouse hepatoma cells were cultured in Dulbecco's modified Eagle medium (DMEM) supplemented with 10% heat-inactivated fetal bovine serum (FBS) and antibiotics. For experimental procedures, the specific cells were treated with the indicated concentration of FeCl_3_ for the indicated time in the presence or absence of FBS.

### Iron staining

RPH were fixed with 10% formalin, and stained with 1% potassium ferrocyanide in 0.5% hydrochloric acid for 15 min to stain Fe^3+^, and followed by washing step with distilled water. Ferrous ion (Fe^2+^) in mitochondria of RPH were visualized by Mito-FerroGreen (Dojindo, Mashikimachi, Japan) according to the manufacturer’s protocol.

### RNA isolation and reverse transcription-quantitative PCR (RT-qPCR)

Total RNA isolation and real-time RT-qPCR were performed as previously described^44,48^. The nucleotide sequence of qPCR primers is listed in Supplementary Table 1. The ΔΔCt method was used to normalize the expression level of target transcripts to TATA-binding protein (Tbp) level^49^. The expression level of the genes in untreated control cells or RPH were set at 1.

### Ultrastructural analysis

Ultrastructural analysis using TEM was performed as described previously^50^. Briefly, the cells were fixed with Karnovsky solution (2% glutaraldehyde/2% paraformaldehyde/0.05 M cacodylate buffer (pH 7.4)) and subsequently post-fixed with buffered 1% osmium tetroxide/1.5% potassium ferrocyanide / 0.05 M cacodylate buffer (pH 7.4), followed by the embedding of cells in epoxy resin. Ultrathin sections were cut and stained with uranyl acetate followed by lead citrate. The stained sections were examined by using a Hitachi H-7650 transmission electron microscope (Hitachi Ltd., Tokyo, Japan).

### Western blot analysis

Western blot analysis was performed as previously described^51^. The immunoreactive proteins were visualized using Chemi-Lumi One Ultra (Nacalai Tesque, Kyoto, Japan) according to the manufacturer’s protocol. Following primary antibodies were purchased from Cell Signaling Technology (Danvers, MA, USA): rabbit polyclonal antibodies against ERK and β-actin, rabbit monoclonal antibodies against AMPKα (23A3) and LC-3B (D11). In addition, rabbit polyclonal antibody against SMAD1 was purchased from Abcam (Cambridge, MA, USA). The immunoreactive proteins were visualized using Chemi-Lumi One Ultra (Nacalai Tesque, Kyoto, Japan) according to the manufacturer's protocol. The luminescence was captured by LAS-4000 mini (GE Healthcare, Tokyo, Japan), and the band intensity was quantified by use of MultiGauge software (GE Healthcare).

### Cell viability assay

Cell viability was assessed by CellTiter-Glo Luminescent Cell Viability Assay Kit (Promega, Madison, WI, USA) according to the manufacturer’s protocol. In this system, ATP content of the living cells can be evaluated by light emission. Amount of luminescence in untreated control cells was set at 100.

### RNA-seq analysis

RPH were treated with different concentration of FeCl_3_ (0, 25, 100, or 400 μM) in the absence of FBS for 2 h (n = 6). After extraction of total RNA, equal amount of RNA was mixed from every treatment, and RNA-seq was performed as described previously^46^.

### Functional category analysis

The genes identified with reads per kilobase of exon per million mapped sequence reads (RPKM) value below 3 using RNA-seq analysis were excluded, which accounted for 5,187 genes. When the r^2^ values for linear and quadratic effects on the expression levels were more than 0.8 against the dose of FeCl_3_, we considered that these genes exhibited dose-dependent changes upon FeCl_3_ treatment in the expression levels, which accounted for 1,448 genes. Moreover, when the ratio of RPKM value of the gene in control cells to that in cells treated with FeCl_3_ (400 μM) was more than 3 or less than one-third, we selected the genes for further analysis. In the end we selected 174 genes that were submitted to DAVID (The Database for Annotation, Visualization and Integrated Discovery^52^: https://david.ncifcrf.gov, accessed October 2019) to further identify functional categories. Functional annotation clustering analysis was performed, and top four clusters that had enrichment score of more than 1 were used in this study. The *P*-value of a gene ontology (GO) term in each cluster was less than 0.05. A summary of the description of each cluster was generated based on the constitutive GO terms.

### Statistical analysis

The experimental data is presented as mean ± SE. The data of relative gene expression was log-transformed to provide an approximation of normal distribution before analysis. Significant differences between the groups in each cell type were analyzed by Student’s *t*-test or one-way analysis of variance (ANOVA). When the group effect in one-way ANOVA was significant, differences among groups were evaluated by Dunnett’s test or Tukey test. As for cell viability assay, effect of FeCl_3_ was evaluated by Dunnett’s test in cells cultured in the presence or absence of FBS. Correlation analysis was performed to examine the dose effect of FeCl_3_ on gene expression. Further, the reciprocal relationship of the log-transformed values of gene transcript level was investigated by means of Pearson's correlation coefficient. *P* < 0.05 was considered to be significant.

## Supplementary information

Supplementary information.
