# Supplementary material for: Response to iron overload in cultured hepatocytes
Source: Sci Rep. 2020 Dec 3;10:21184. doi: 10.1038/s41598-020-78026-6 (PMC7713074; doi:10.1038/s41598-020-78026-6)
Supplement: Supplementary file 1 — Supplementary information. [file 41598_2020_78026_MOESM1_ESM.docx]

Supplementary information

Response to iron overload in cultured hepatocytes

Hsuan-Ju Chen^1^, Makoto Sugiyama^2^, Fumie Shimokawa^3^, Masaru Murakami^3^, Osamu Hashimoto^4^, Tohru Matsui^1^, and Masayuki Funaba^1^*

^1^Division of Applied Biosciences, Graduate School of Agriculture, Kyoto University, Kyoto 606-8502, Japan

^2^Laboratory of Veterinary Anatomy, Kitasato University School of Veterinary Medicine, Towada 034-8628, Japan.

^3^Laboratory of Molecular Biology, Azabu University School of Veterinary Medicine, Sagamihara 252-5201, Japan

^4^Laboratory of Experimental Animal Science, Kitasato University School of Veterinary Medicine, Towada 034-8628, Japan.

*Corresponding author: Masayuki Funaba, Ph.D.

Division of Applied Biosciences

Graduate School of Agriculture

Kyoto University

Kitashirakawa Oiwakecho, Kyoto 606-8502, Japan

Tel.: +81-75-753-6055

Fax: +81-75-753-6344

E-mail: funaba.masayuki.8w@kyoto-u.ac.jp


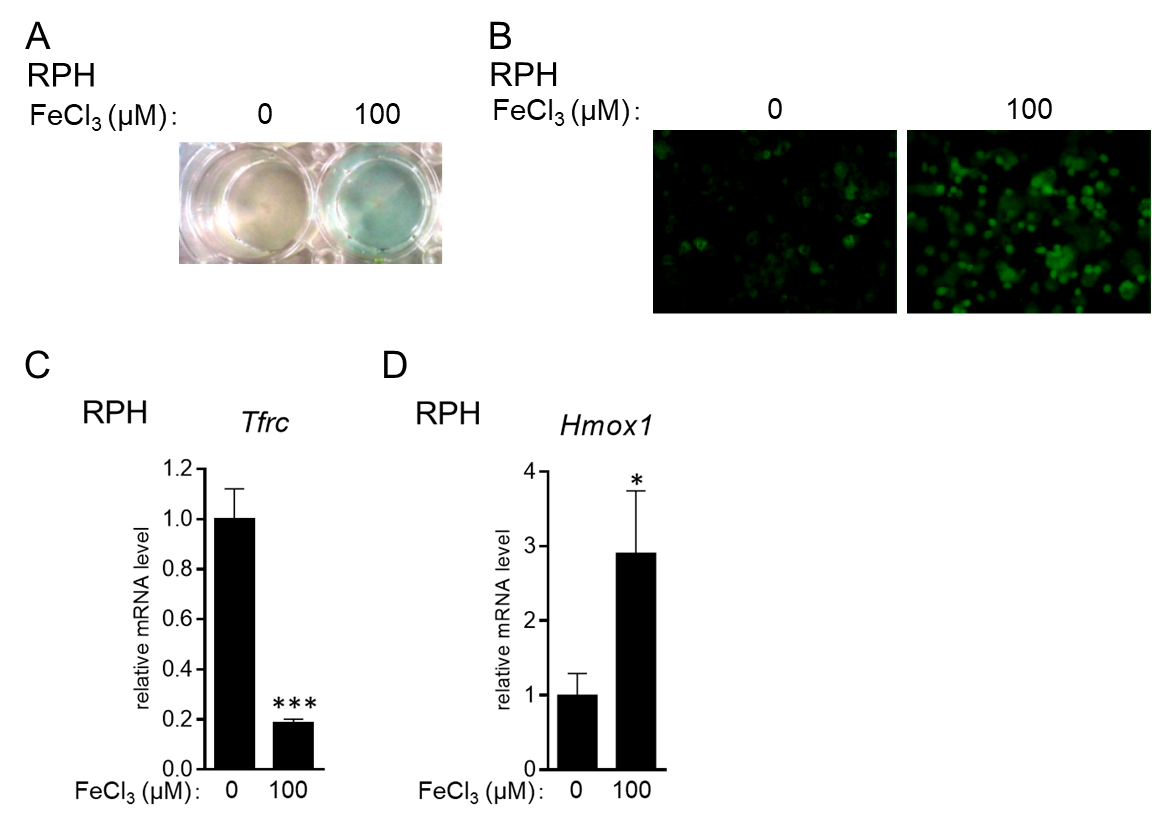


Figure S1. Treatment with FeCl_3_ induces iron accumulation in RPH

RPH were treated with FeCl_3_ (0 or 100 μM) for 16 h or 24 h. (A) Berlin blue staining and (B) Mito-FerroGreen staining were performed. (C and D) Expression level of *Tfrc* or *Hmox1* genes was examined by RT-qPCR analysis, and the expression level in the untreated control cells were set at 1. * and *** indicate *P* < 0.05 and *P* < 0.001, respectively.


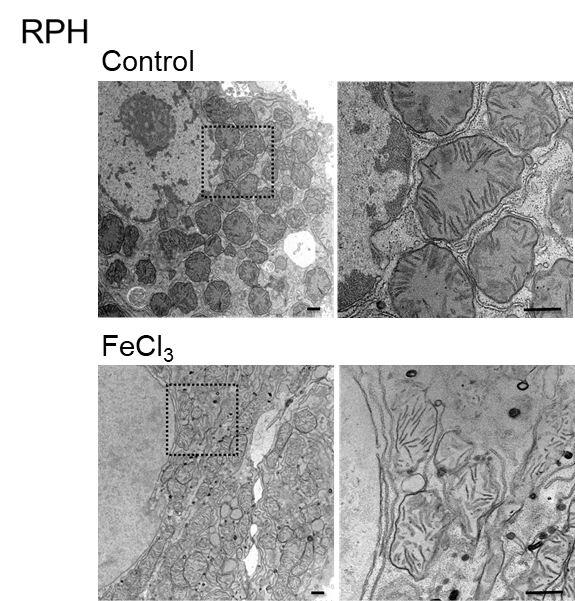


Figure S2. Emergence of granules with high density of electron concentration in FeCl_3_-treated RPH

RPH were treated with FeCl_3_ (0 or 100 μM) for 8 h. Ultrastructure of RPH was analyzed by transmission electron microscopy. Representative image depicting around mitochondria (*left panel*), and higher magnification of the dotted line square (*right panel*). The bar in the image is indicative of 500 nm.


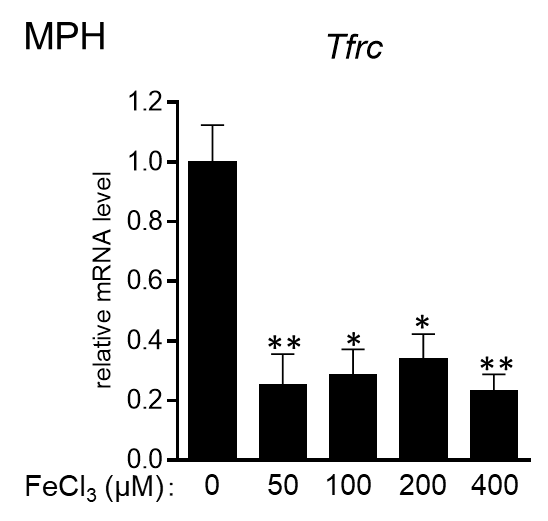


Figure S3. Down-regulation of *Tfrc* in MPH treated with FeCl_3_

MPH were treated with the indicated concentration of FeCl_3_ for 24 h. Expression level of *Tfrc* gene was examined by RT-qPCR analysis, and the expression level in the untreated control cells were set at 1. * and ** indicate *P* < 0.05 and *P* < 0.01, respectively.


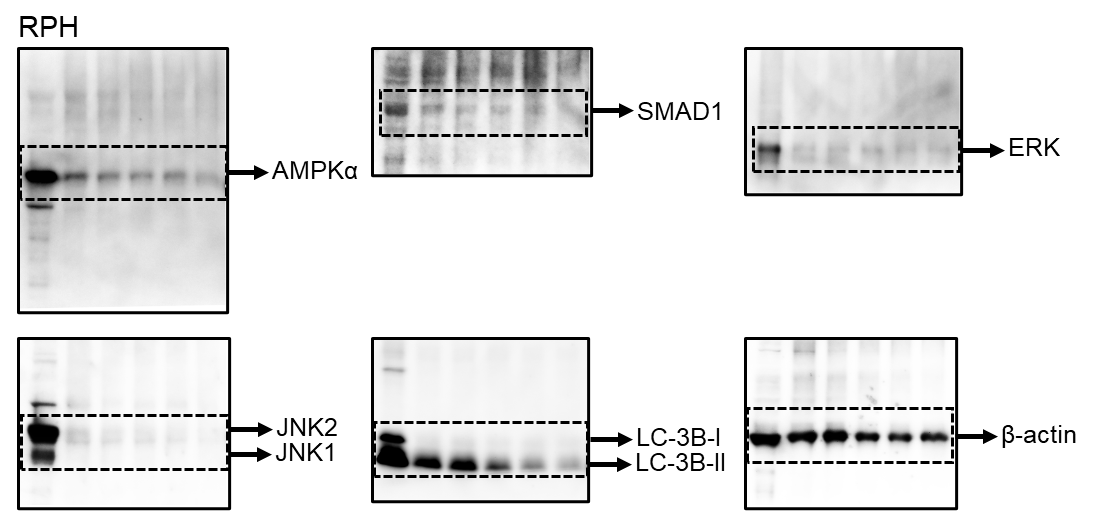


Figure S4. Images of full-length blot shown in Fig. 1A

Western blot analysis was visualized by chemical luminescence-based method. The raw results of Western blot analysis are shown. Dashed squares were cropped and shown in Fig. 1A.

Figure S5. Intensity of band shown in Fig. 1A

The band intensity of molecules shown in Fig. 1A was quantified, and the ratio of the band intensity of β-actin was calculated. The relative ratio in cells treated without FeCl_3_ was set at 100.
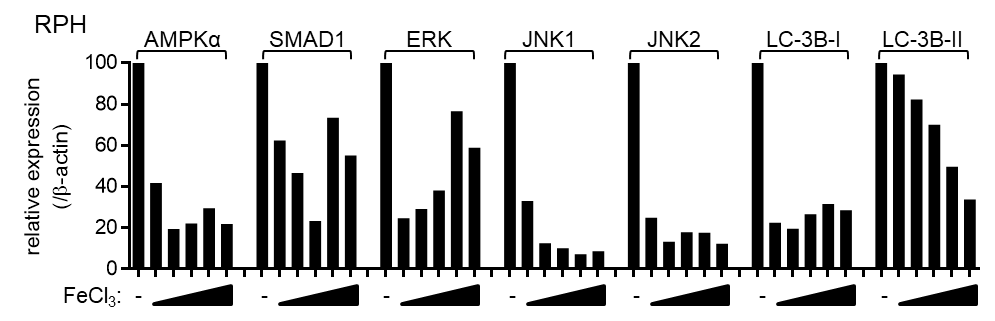


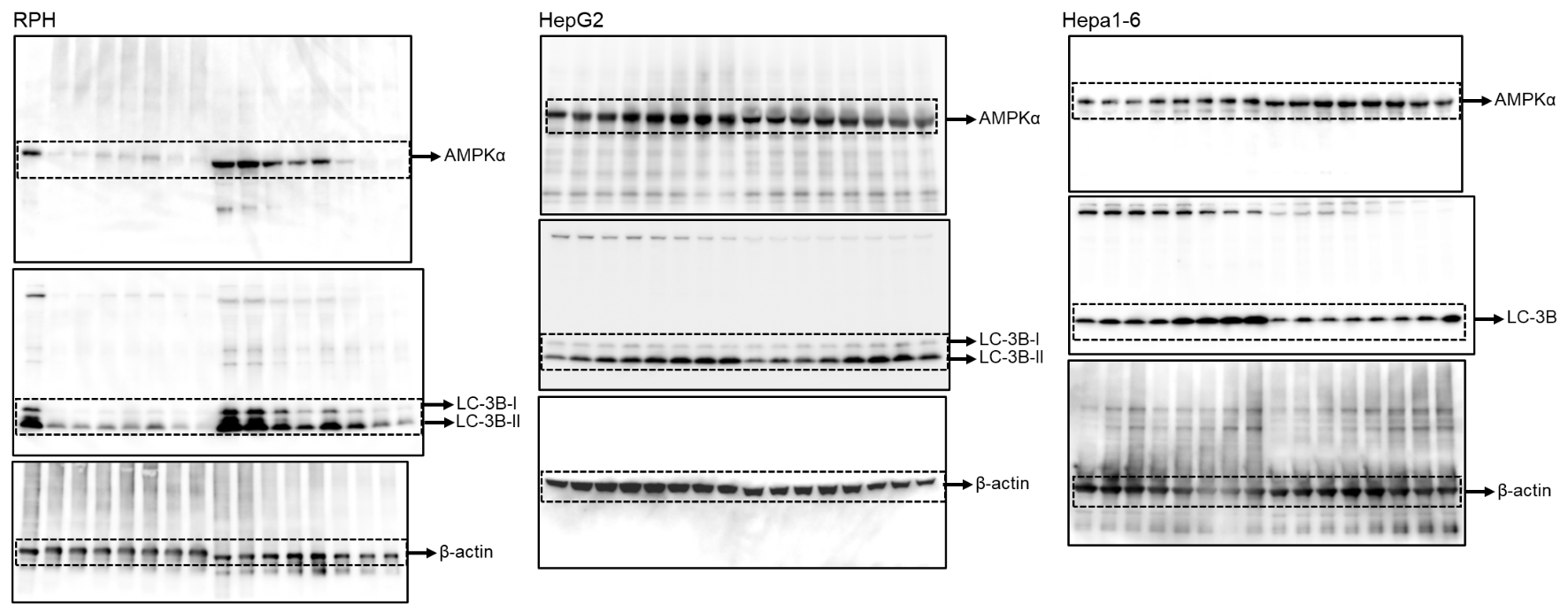


Figure S6. Images of full-length blot shown in Fig. 4B

Western blot analysis was visualized by chemical luminescence-based method. The raw results of Western blot analysis are shown. Dashed squares were cropped and shown in Fig 4B.


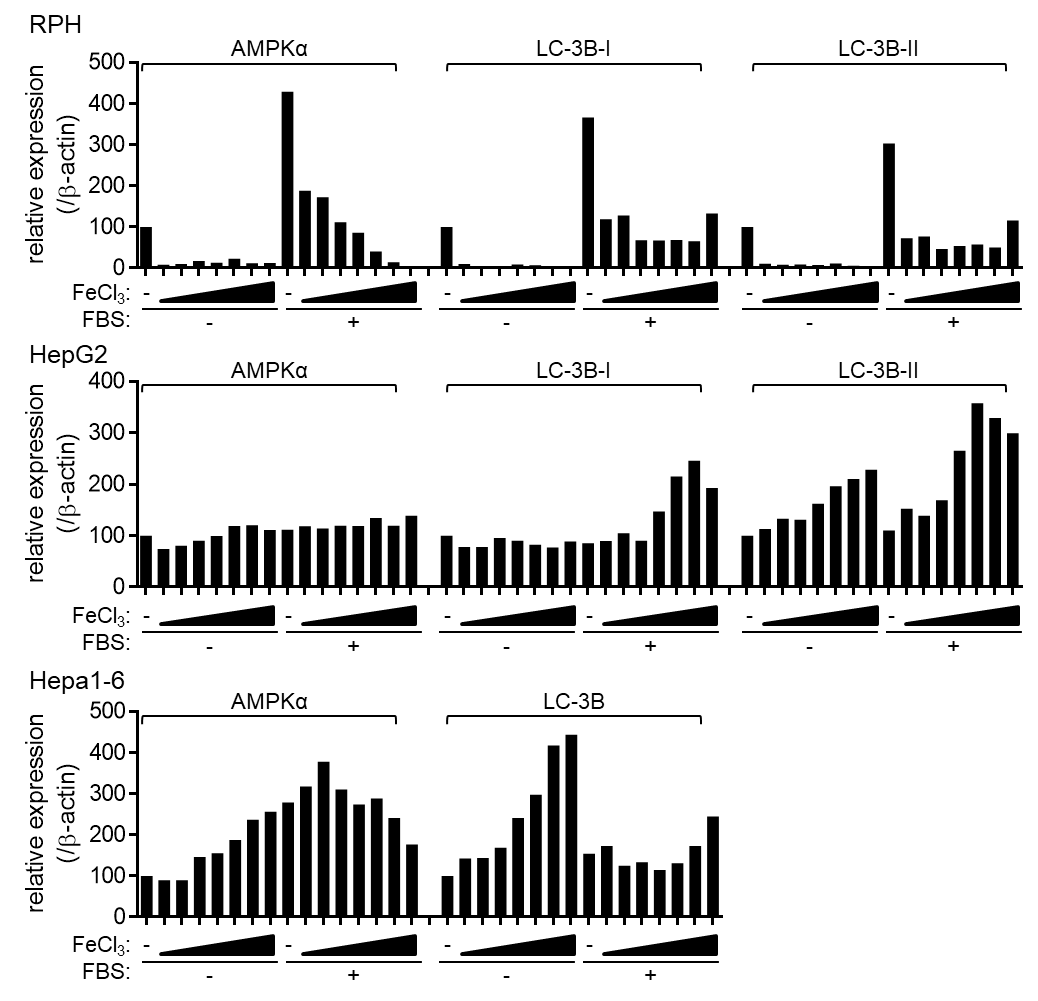


Figure S7. Intensity of band shown in Fig. 4B

The band intensity of molecules shown in Fig. 4B was quantified, and the ratio of the band intensity of β-actin was calculated. The relative ratio in respective cells treated without FeCl_3_ in the absence of FBS was set at 100.


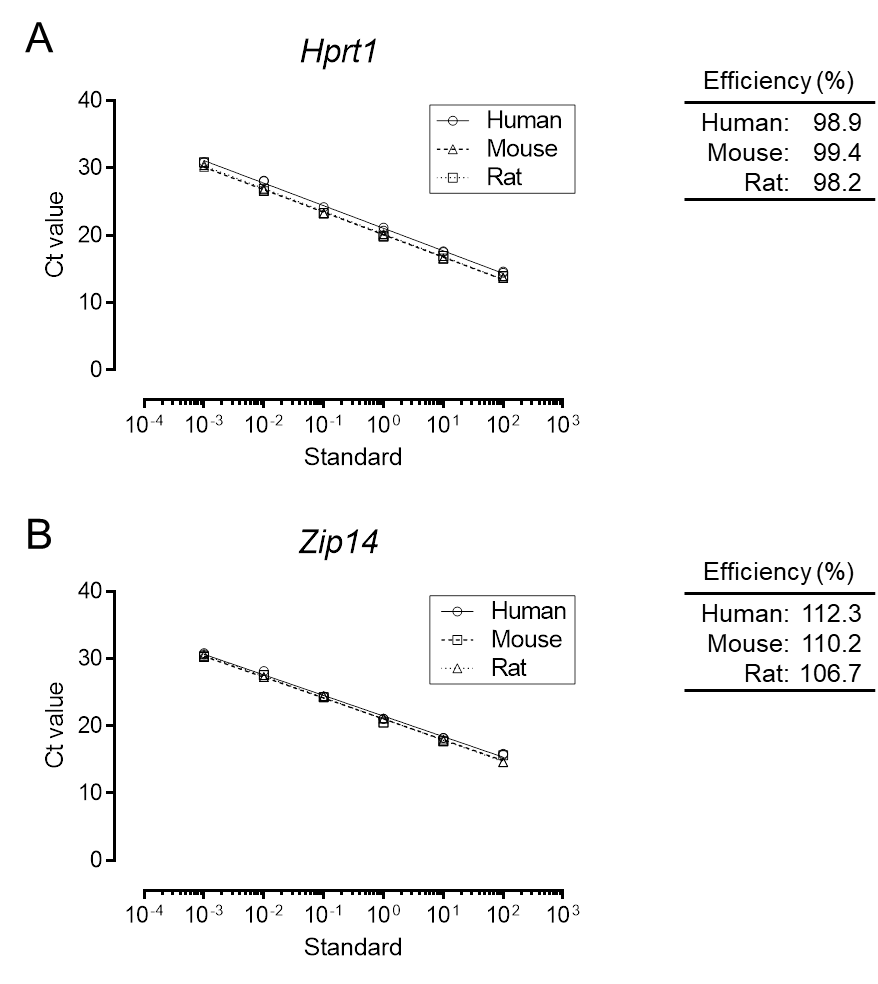


Figure S8. Amplification of genes using common primers for human, mouse, and rat

PCR primers to detect *Hprt1* and *Zip14* genes were designed. PCR was performed using the plasmids containing the respective genes from human, mouse, or rat as the template. Amplification of (A) *Hprt1* and (B) *Zip14* is plotted and efficiency of amplification is shown.

Supplemental Table 1. Oligonucleotide PCR primers for RT-qPCR

5’-primer 3’-primer

Rat

*Atg5* 5’-CTGTTCGATCTTCTTGCATCAAGC-3’ 5’-TCCTTTTCTGGAAAACTCTTGAAATGTAC-3’

*Chac1* 5’-TTATTGCACCATGTTGGTGTG-3’ 5’-TTCTGGTTGGTACCAGAGGAC-3’

*Dcytb* 5’-CTGCACAGCTGGGTTGGACTGA-3’ 5’-GCGGAGCCCATGGAAGCAGAAAGA-3’

Ern1 5’-GGTGGCGTTCATCATCACTT-3’ 5’-GAGCTGAATTTTCTCCAGTTCC-3’

*Fgfr2* 5’-TGTGCACAAGCTGACCAAGCG-3’ 5’-TGTTGGAGTTCATGGACGAGCTG-3’

*Gabpa* 5’-TGCACTGGAAGGCTACAGAAAAGAG-3’ 5’-GGTCTGTGGACCACTGTATAGGAT-3’

*Hes6* 5’-GAGGTGCTGGAGCTGACCGTGC-3’ 5’-GATGTAGCCAGCAGCGAAGCGCTCG-3’

*Hprt1* 5’-GACCGGTTCTGTCATGTCG-3’ 5’-ACCTGGTTCATCATCACTAATCAC-3’

*Jnk1* 5’-TGTGGAATCAAGCACCTTCACTCTG-3’ 5’-TGCCAGTCCAAAATCAAGAATCTTCAAA-3’

*Mrpl54* 5’-GGGTCAACATCTACAAGGAAGG-3’ 5’-GTTCTAGGTCCTCTAGCTTTTTGG-3’

*Mrps24* 5’-TACATCGCACACCGAAAGGGCT-3’ 5’-GGAATACATCCTCCAGGGTTCTC-3’

*Nrbf2* 5’-TGGAAGGACCCCTCAACCTT-3’ 5’-TTTTGTGACAAGAAATCGCTTC-3’

*Ppp6c* 5’-CGCAGGTGTACGGATTTTATGATG-3’ 5’-TGAGCATGTCAAAAACTTTGGTACAG-3’

*Ppp6r1* 5’-GAGAGCATCCCAGGCAAGCCA-3’ 5’-CCTCCTCCTCGCTGTCTGTGC-3’

*Slc1a4* 5’-GGGGCTGGAGAACTCAGAACCT-3’ 5’-CAAGATTGGAAGGGAACAGGTTTCT-3’

*Slc16a7* 5’-CAATGACATCAAGGACATTTTCAAGAC-3’ 5’-CAACACACTACTGATGGGACCTC-3’

*Slc30a9* 5’-CAAACTCCGAGAACTGACAAAG-3’ 5’-GGAGCTTTGAGTTCTGCACCT-3’

*Tfam* 5’-TAAAGCTAAACACCCAGATGCAAA-3’ 5’-ACTCAGCTTTAAAATCCGCTTCATA-3’

*Tmem5* 5’-AATAGAGCATCGTGTGCAAATCTGG-3’ 5’-GCGAGAGTGCCTTCAAAAACAT-3’

*Tmem120a* 5’-CTCTCTCCCATCCGAGTCCCTG-3’ 5’-CAGAACCAGACTCAGGTACAACCC-3’

*Tmem126a* 5’-GTGACACAAGCTCGAGTAGCA-3’ 5’-TCGTACAGATTTCACAGTTCAAATTA-3’

*Tnfrs1b* 5’-GCCGGCTCCAGGAGTTCAGATTC-3’ 5’-TGGCTTGGGAAGAACACTGAGAGC-3’

*Zip14* 5’-TAACGCACTTTTCCAGCTCATC-3’ 5’-CCCAAAGACCACAGCAGACTTGGA-3’

Human-Mouse-Rat

*Dmt1* 5’-TGTCACCGTCAGTATCCCAAG-3’ 5’-ATGGCTGAGCCAATGACTTC-3’

*Fpn* 5’-GTCATTGCTGCTAGAATCGG-3’ 5’-CATTTTCTTGCAGCAACTGTGTC-3’

*Fth1* 5’-GCCATCAACCGCCAGATCAACCT-3’ 5’-TGGTTCTGCAGCTTCATCAGTTTCTC-3’

*Hmox1* 5’-TGAAGGAGGCCACCAAGGAGGT-3’ 5’-GGTACAAGGAGGCCATCACCAGCTT-3’

*Hprt1* 5’-ATTCCTATGACTGTAGATTTTATCAGCTGAAGAGCTA-3’ 5’-CCAGTTAAAGTTGAGAGATCATCTCCACCAA-3’

*Ptgs2* 5’-TCCAACCTCTCCTACTACACCAG-3’ 5’-TCCAGAACTTCTTTTGAATCAGG-3’

*Steap3* 5’-CCTRCAGGCTGGCCCAAGGGATGG-3’ 5’-GCTGASGCCAGGGATCCCATGTCCA-3’

*Steap4* 5’-TTGGGAATCACTTCYTTGCCATC-3’ 5’-CAGGGTCARATAACCCAGTTTGGA-3’

*Zip14* 5’-CAGGAACCTCTCCACGTGCTTTAG-3’ 5’-ATGGTGGGGCAGAACTCCTGGA-3’

*Tfrc* 5’-CAAGCYAGATCAGCATTCTCTAACTTGTTT-3’ 5’-AGCMAGTTTCATCTCCACATGACTGTTATC-3’
